# Successful Cases of Resection of the Ribs

**Published:** 1828-12

**Authors:** M. Cittadini


### RESECTION OF THE RIBS.

*Successful Cases of Resection of the Ribs.* By M. CITTADINI.

AMONG the operations which have enriched modern surgery, is the resection of the ribs, first made known to the practitioners of Great Britain by one, which was unsuccessful, performed by RICHERAND, although the same had been previously crowned with success by CITTADINI. From the following narratives, taken from the Annali Universali di Medicina, it will be found to be unattended with the danger usually anticipated. Hemorrhagy from the intercostal artery may be easily suppressed near the sternum, and the ligature is only required when the middle and posterior parts of the rib are removed.

The first of the following cases was published in the Journal Complementaire des Sciences Medicales, in the year 1820, two years after the case of Richerand, although it was performed in 1818. It has been deemed advisable to reproduce it in this place, for the purpose of presenting the whole body of evidence in one focus.

CASE I.—A woman had fistulous ulcers in the left breast, of long standing. Caustics and repeated incisions were employed, without effect. The sternum and cartilages of the sixth and seventh ribs were found, by the introduction of a probe, to be carious, which, on laying bare the parts by incisions, was seen to be upwards of an inch in length; and the cartilages were swoln and perforated in many places for the space of three inches. The actual cautery was employed to promote exfoliation of the bone, without effect, and excited inflammation of the pleura.

Six months afterwards, the pus made its way into the chest, causing continual pain, great difficulty of breathing, and great emaciation.

The resection of the ribs being resolved upon, all the diseased surface of the bone was laid bare, when an opening into the cavity of the thorax was found between the sixth and seventh ribs. The intercostal muscles were divided; their arteries tied by means of a blunt needle, and the two diseased ribs were cut through on the inside of the ligatures. A large trephine was applied on the sternum, so as to include the diseased portion, which was finally separated from the subjacent pleura by means of a spatula. The proximity of the internal mammary artery prevented the removal of this membrane, although its unhealthy condition seemed to indicate the necessity of it.

The introduction of air through the wound into the thorax produced great fear of suffocation, but the wound was promptly covered by lint spread with cerate. Stimulants, frictions, and insufflations of air into the lungs, were required to revive the patient, whose life was nearly extinct.

During the two succeeding months, the difficulty of breathing was very great; but, as soon as the wound was cicatrised, the symptoms disappeared, and the patient was restored to perfect health.

Case II.—The cure of a fungous tumor seated on the cartilages of the sixth, seventh, and eighth ribs, near the xiphoid cartilage, had been often ineffectually tried, by cautery and the knife, when M. Cittadini, thinking it to be rooted in the rib, removed the whole of the ligaments around the tumor. He cut through some fibres of the rectus and obliquus, so as to expose the whole of the cartilages, and discovered that the diseased parts occupied a space whose diameter was about two inches. The cartilages were then cut through by a strong bistoury; the diseased portion was raised by a spatula, when it was discovered that it strongly adhered by its internal surface to a fungous mass within, whose laceration occasioned an abundant hemorrhagy. The flow of blood was arrested by the actual cautery, and the cicatrization was completed at the end of three weeks, without any unfavorable symptom.

Case III.—The cartilages of the sixth rib and part of the bone were carious, at the bottom of a fistulous opening in the integuments. The latter were dissected off, and the diseased portion of the cartilage and bone was about an inch and a half long. The cartilage was cut through by the bistoury, and the bone divided by cutting pincers. The principal arteries were tied, the smaller compressed, and the excised portion was removed with the usual circumspection; the pleura having been opened during the operation in several places. The respiration was at first short and laborious, but at the expiration of some hours returned to its natural state.

The wound was cicatrised at the end of two months, and the patient perfectly cured, without having experienced any symptom of affection in the chest.

Case IV.—After violent pleurisy, a tumor, which was hard and painful on pressure, appeared near the sternal articulation of the sixth rib. It slowly suppurated and opened externally, leaving a fistulous ulceration. The diseased bone was exposed and removed The subjacent and thickened pleura was cut in several places An abundant hemorrhage flowed from the divided intercostal which nevertheless was stopped by compression.

The cure in this case was not completed for six months.

Case V.—A fistulous sinus terminated in a carious point in the upper edge of the third rib, arising from a blow. The lower part of the bone, being sound, was preserved; and the patient was cured in two months.

---

